# A territory-wide study on adults hospitalized for rhinovirus infection – mortality, severe adverse respiratory and renal outcomes and relationships with multiple comorbidities

**DOI:** 10.1099/jmm.0.002194

**Published:** 2026-10-07

**Authors:** Wang Chun Kwok, Isaac Sze Him Leung, James Chung Man Ho, Chun Ka Wong, Zhiling Yuan, Desmond Yat Hin Yap

**Affiliations:** 1 Division of Respiratory Medicine, Department of Medicine, The University of Hong Kong, Queen Mary Hospital, Hong Kong, PR China; 2 Department of Statistics, The Chinese University of Hong Kong, Shatin, New Territories, Hong Kong, PR China; 3 Division of Cardiology, Department of Medicine, The University of Hong Kong, Queen Mary Hospital, Hong Kong, PR China; 4 Department of Medicine, School of Clinical Medicine, Li Ka Shing Faculty of Medicine, The University of Hong Kong, Hong Kong, PR China; 5 Division of Nephrology, Department of Medicine, The University of Hong Kong, Queen Mary Hospital, Hong Kong, PR China

**Keywords:** acute kidney injury, Charlson comorbidity index, hospital-acquired pneumonia, respiratory failure, rhinovirus

## Abstract

**Introduction.** While rhinovirus was thought to cause mild infections such as the common cold, severe infections such as community-acquired pneumonia and asthma exacerbations can also occur. Children with chronic medical conditions have been shown to have increased risks of severe infections.

**Hypothesis/Gap statement.** There is a difference in the risks of serious in-hospital outcomes among adults hospitalized for rhinovirus or influenza virus infection. Multiple morbidities in adult patients are associated with severe infections among patients hospitalized for rhinovirus infections.

**Aim.** To compare the risks of serious in-hospital outcomes among adults hospitalized for rhinovirus or influenza virus infection and assess the association between multiple morbidities as represented by Charlson comorbidity index (CCI) in predicting risks of severe infections among patients hospitalized for rhinovirus infections.

**Methodology.** We conducted a territory-wide retrospective study on adults hospitalized for rhinovirus or influenza virus infection between 1 January 2016 and 30 June 2023 in Hong Kong. The in-patient mortality, severe respiratory failure (SRF), secondary bacterial pneumonia and acute kidney injury (AKI) were compared. The association between multi-morbidities as reflected by CCI and severe in-hospital outcomes among adult patients hospitalized for rhinovirus infections was also assessed.

**Results.** A total of 41,206 and 2,222 patients were hospitalized for influenza and rhinovirus infections, respectively. Patients with rhinovirus infection showed a significantly higher risk of SRF, secondary bacterial pneumonia and AKI compared to those with seasonal influenza (*P*<0.001 for all). Higher CCI (as both continuous variables, by grading as well as cut-off identified by receiver operating characteristic curve analysis) was associated with increased risks of severe in-hospital outcomes.

**Conclusion.** Adults hospitalized for rhinovirus infection were associated with a significantly increased risk of adverse respiratory and kidney outcomes compared with those with seasonal influenza. More medical comorbidities, as reflected by CCI, were shown to be associated with an increased risk of severe in-hospital outcomes.

## Data Summary

All available data is presented in the article and the supplementary material, and no additional data is available.

## Introduction

As a member of the picornavirus family, rhinovirus is a small (30 nanometre) single-stranded RNA virus [1, 2]. In the past, rhinovirus was commonly neglected because it was generally perceived as a respiratory virus capable only of causing mild common cold [3]. However, apart from asymptomatic infections and common cold, it can also result in community-acquired pneumonia [4, 5] and asthma exacerbations [6].

In the paediatric population, rhinovirus has been reported to be frequently associated with asthma exacerbations and lower respiratory tract infection. It has also been thought to be a potentially important contributor to hospitalization in children [7]. In adults, there have also been reports from single centres suggesting that rhinovirus infection in adults was associated with significantly higher mortality and longer hospitalization when compared with influenza virus infection. Institutionalized older adults were at risk for severe rhinovirus infection, while higher Charlson comorbidity index (CCI) scores were associated with increased mortality risks [8]. These findings suggest that rhinovirus infection, especially among hospitalized patients, should not be considered mild and self-limiting. Rhinovirus infection can result in life-threatening infections in patients who have co-existing diseases, particularly those with asthma.

However, there has been a lack of evidence on how to properly stratify the risks of rhinovirus infection among adult patients. To facilitate clinical management and risk stratification, adopting an easy-to-use and well-established scoring system will be helpful. The CCI, which consisted of 19 items corresponding to different medical comorbid conditions displaying different clinical weights, was initially developed to predict the risk of 1-year mortality, controlling for severity of illness, in patients admitted to a general internal medicine service, which subsequently was used to predict mortality of 685 patients with breast cancer from comorbid disease over 10 years [9, 10]. It was later used in different populations as a prognostic measure in longitudinal studies to predict mortality. The CCI was also demonstrated to be a scoring system to predict outcomes for coronavirus disease 2019 (COVID-19) [9, 11, 12].

Given prior reports that mortality risk in adult rhinovirus infection is related to CCI, it is worthwhile to assess the association between CCI and various severe in-hospital outcomes among adult patients hospitalized for rhinovirus infection.

## Methods

### Study design and participants

This was a territory-wide retrospective study that aimed to compare mortality and serious in-hospital outcomes in adult patients hospitalized for rhinovirus and seasonal influenza infections. We then examined the associations between multi-morbidity, as reflected by the CCI, and serious clinical outcomes in adult patients hospitalized for rhinovirus infection in the second stage of the study. Adult patients who were admitted to public hospitals in Hong Kong for rhinovirus infection and seasonal influenza between 1 January 2016 and 30 June 2023 were included. The index admission was defined as the first admission for rhinovirus within the study period, with the index date defined as the date of admission. Any subsequent admission for rhinovirus during the study period would not be counted. Patients were identified from the Clinical Data Analysis and Reporting System (CDARS) of Hospital Authority (HA) using the International Classification of Diseases (ICD), ninth revision codes of 487.8 for seasonal influenza and 079.3 for rhinovirus infections. CDARS is an electronic health record database managed by the HA, which is a public healthcare service provider that has covered more than 90% of the Hong Kong population since 1993 [13–15]. The included patients had laboratory-confirmed influenza or rhinovirus infections by multiplex PCR testing of respiratory specimens. Patients who had co-infections with other respiratory viruses (including influenza, parainfluenza, adenovirus, human metapneumovirus, severe acute respiratory syndrome coronavirus 2 or respiratory syncytial virus) as detected by multiplex PCR were excluded.

Regarding vaccination status, those who received seasonal influenza vaccine within the 12-month period prior to the index admission were considered vaccinated. Those who received pneumococcal vaccine within the 5-year period prior to the index admission were also considered vaccinated.

Influenza was chosen to be the comparator group, as it is the most common respiratory virus causing hospitalized infections in Hong Kong. Influenza is also reported to have significant risks of mortality and serious in-hospital outcomes in adult patients, which serves as the rationale for vaccination strategies against the influenza virus.

### Outcome measurements

The first main exposure of interest was rhinovirus and seasonal influenza. The main outcomes of interest were: (1) death during hospitalization, (2) severe respiratory failure (SRF) requiring invasive or non-invasive mechanical ventilation, (3) secondary bacterial pneumonia and (4) acute kidney injury (AKI). Secondary bacterial pneumonia was identified by ICD-9 diagnostic codes 481 or 482 or by the growth of pathogenic organisms in respiratory specimens during the same admission episode. We first compared the incidence rates of the above outcomes between patients hospitalized for seasonal influenza and rhinovirus infections. SRF was defined as the use of high-flow nasal cannula, non-invasive and invasive ventilation using the procedure code of 93.9× and 96.7×. AKI was defined according to the RIFLE criteria, and estimated glomerular filtration rate (eGFR) criteria were adopted in the current study [16]. The baseline eGFR was the eGFR recorded in the outpatient setting prior to the index admission, with no missing data from the cohort. The RIFLE criteria classified renal outcomes as follows: Risk (R): serum creatinine increased by 1.5 times or eGFR decreased by >25%, urine output <0.5 ml kg^−1^ h^−1^ for 6 h. Injury (I): serum creatinine increased by two times or eGFR decreased by >50%, urine output <0.5 ml kg^−1^ h^−1^ for 12 h. Failure (F): serum creatinine increased by three times or eGFR decreased by >75%, urine output <0.3 ml kg^−1^ h^−1^ for 24 h or anuria for 12 h. Loss (L): persistent AKI with complete loss of kidney function for more than 4 weeks. End-stage (E): end-stage renal disease (requiring dialysis for more than 3 months).

Next, we explored the association between multi-morbidity, as reflected by the CCI, and the development of severe in-hospital outcomes among patients with rhinovirus infections.

### Statistical analysis

Descriptive tables were created to present the incidence rates of severe in-hospital outcomes stratified by seasonal influenza and rhinovirus infections, with demographic and clinical data described in actual frequency or mean±sd, or median (interquartile range), where appropriate. Baseline demographic and clinical data were compared between patients with seasonal influenza and rhinovirus using an independent t-test or Mann–Whitney U test, where appropriate. To compare the risk of mortality and severe in-hospital complications between patients hospitalized with seasonal influenza and rhinovirus infections, we first performed univariate logistic regression analyses followed by multivariable analysis. The covariates adjusted in multivariable analyses included age, sex, CCI, presence of diabetes mellitus (DM), chronic airway diseases, cardio-/cerebro-vascular diseases and previous vaccination against influenza and pneumococcal polysaccharide and conjugated vaccine.

To better control for the potential confounders, propensity score adjustment was performed. The propensity score was estimated using a logistic regression model including age, sex, CCI, presence of DM, chronic airway diseases, cardio-/cerebro-vascular diseases and previous vaccination against influenza and pneumococcal polysaccharide and conjugated vaccines. Patients were matched at a 2 : 1 ratio using nearest-neighbour matching without replacement, with a calliper width of 0.3 sd of the logit of the propensity score. Covariate balance before and after matching was assessed using standardized mean differences, with values below 0.1 considered indicative of acceptable balance.

The association between multi-morbidity, as reflected by CCI, and the development of severe in-hospital outcomes among patients with rhinovirus infections was assessed first by univariate and then multivariable analyses. The CCI was assessed as a continuous variable, a categorical variable (0: no comorbidity, 1–2: mild, 3–4: moderate, ≥5: severe) and using the cut-off determined by receiver operating characteristic (ROC) curve analysis. In ROC analysis, the CCI that has the largest Youden’s index is the optimal cut-off. The covariates adjusted in the multivariable analyses included sex, presence of chronic airway diseases and previous vaccination against influenza and pneumococcal polysaccharide and conjugated vaccines. These covariates were chosen as they were factors well reported to be associated with severe respiratory tract viral infections,

Multi-collinearity of the factors included in multivariable analyses was tested and presented in Table S1 (available in the online Supplementary Material).

Subgroup analyses were performed among patients aged <65 years and ≥65 years.

Data analyses were performed using R version 4.0.3 software (R Core Team, Vienna, Austria). For all statistical analyses, statistical significance was assessed at an α level of 0.05. STROBE and RECORD reporting guidelines were followed in the generation of this report.

### Ethical considerations

The study was approved by the Institutional Review Board (IRB) of the University of Hong Kong and HA Hong Kong West Cluster (UW 24–137). Patient informed consent was waived in this retrospective study by the IRB, as it is a retrospective study without active patient recruitment and the data were already de-identified. The study was conducted in compliance with the Declaration of Helsinki.

## Results

### Patients’ characteristics

A total of 41,206 and 2,222 adult patients were hospitalized for seasonal influenza and rhinovirus infections, respectively, to public hospitals in Hong Kong during the period from 1 January 2016 to 30 June 2023 (Table 1). Patients with rhinovirus infections were older and had more comorbidities than those with seasonal influenza (Table 1).

**Table 1.** Baseline characteristics of patients hospitalized with seasonal influenza or rhinovirus infection before and after propensity score matching

	All	Unmatched				Matched			
		**Influenza virus**	**Rhinovirus**	***P* value**	**SMD**	**Influenza virus**	**Rhinovirus**	***P* value**	**SMD**
Number of subjects (*N*)	43,428	41,206	2,222			3,417	1,872		
Age, years (mean±sd)	67.5±19.9	67.36 (19.84)	70.69 (20.22)	<0.001	0.166	69.96 (19.41)	69.69 (20.34)	0.636	0.014
Male [*N*, (%)]	22,064 (50.8)	20,336 (49.4)	1,411 (63.5)	<0.001	0.086	1,788 (52.3)	1,014 (54.2)	0.210	0.037
eGFR, ml min^−1^/1.73 m^2^ (mean±sd)	65.4±26.1	65.1±26.4	69.3±30.0	<0.001	0.141	58.7 (25.6)	71.4 (29.4)	<0.001	0.458
CCI group [*N*, (%)]				<0.001	0.306			0.810	0.019
0	6,816 (15.7)	6,612 (16.0)	204 (9.2%)			375 (11.0)	204 (10.9)		
1–2	7,461 (17.2)	7,216 (17.5)	245 (11.0)			431 (12.6)	225 (12.0)		
≥3	29,151 (67.1)	27,378 (66.4)	1,773 (79.8)			2,611 (76.4)	1,443 (77.1)		
Malignancies [*N*, (%)]	3,136 (7.2)	2,780 (6.7)	356 (16.0)	<0.001	0.295	440 (12.9)	265 (14.2)	0.205	0.037
Diabetes mellitus [*N*, (%)]	10,541 (24.3)	10,061 (24.4)	480 (21.6)	0.003	0.067	740 (21.7)	389 (20.8)	0.478	0.021
Asthma [*N*, (%)]	2,519 (5.8)	2,219 (5.4)	300 (13.5)	<0.001	0.280	405 (11.9)	225 (12.0)	0.893	0.005
Bronchiectasis [*N*, (%)]	1,093 (2.5)	993 (2.4)	100 (4.5)	<0.001	0.115	134 (3.9)	82 (4.4)	0.463	0.023
COPD [*N*, (%)]	4,423 (10.2)	3,965 (9.6)	458 (20.6)	<0.001	0.310	630 (18.4)	373 (19.9)	0.199	0.038
Acute myocardial infarction [*N*, (%)]	7,035 (16.2)	6,611 (16.0)	424 (19.1)	<0.001	0.080	588 (17.2)	329 (17.6)	0.765	0.010
Heart failure [*N*, (%)]	5,846 (13.5)	5,348 (13.0)	498 (22.4)	<0.001	0.249	655 (19.2)	368 (19.7)	0.693	0.012
Peripheral vascular disease [*N*, (%)]	464 (1.1)	432 (1.0)	32 (1.4)	0.206	0.035	40 (1.2)	23 (1.2)	0.957	0.005
Stroke [*N*, (%)]	6,684 (15.4)	6,259 (15.2)	425 (19.1)	<0.001	0.105	544 (15.9)	304 (16.2)	0.792	0.009
Dementia [*N*, (%)]	1,741 (4.0)	1,176 (2.9)	565 (25.4)	<0.001	0.685	578 (16.9)	352 (18.8)	0.092	0.049
Connective tissue disease [*N*, (%)]	1,087 (1.3)	555 (1.3)	532 (23.9)	<0.001	0.723	403 (11.8)	293 (15.7)	<0.001	0.112
Peptic ulcer [*N*, (%)]	1,459 (3.4)	1,325 (3.2)	134 (5.1)	<0.001	0.094	142 (4.2)	88 (4.7)	0.390	0.027
Chronic liver disease [*N*, (%)]	938 (2.2)	892 (2.2)	46 (2.1)	0.823	0.007	65 (1.9)	38 (2.0)	0.828	0.009
Hemiplegia [*N*, (%)]	510 (1.2)	458 (1.1)	52 (2.3)	<0.001	0.265	0 (0.0)	0 (0.0)		<0.001
Leukaemia [*N*, (%)]	253 (0.6)	213 (0.5)	40 (1.8)	<0.001	0.120	33 (1.0)	25 (1.3)	0.273	0.035
Lymphoma [*N*, (%)]	246 (0.6)	208 (0.5)	38 (1.7)	<0.001	0.115	31 (0.9)	27 (1.4)	0.099	0.050
HIV infection [*N*, (%)]	61 (0.1)	54 (0.1)	7 (0.3)	0.049	0.039	11 (0.3)	7 (0.4)	0.949	0.009
Influenza vaccine [*N*, (%)]	13,386 (30.8)	12,557 (30.5)	829 (37.3)	<0.001	0.145	1,276 (37.3)	685 (36.6)	0.609	0.016
Pneumococcal conjugate vaccination [*N*, (%)]	4,988 (11.5)	4,698 (11.4)	290 (13.1)	0.019	0.050	415 (12.1)	246 (13.1)	0.315	0.030
Pneumococcal polysaccharide vaccination [*N*, (%)]	1,649 (3.8)	1,541 (3.7)	108 (4.9)	0.008	0.055	148 (4.3)	89 (4.8)	0.521	0.020

COPD, Chronic obstructive pulmonary disease; HIV, Human immunodeficiency virus; SMD, Standardized mean difference.

### Severe in-hospital outcomes among influenza and rhinovirus patients

#### Whole cohort

Adults who were hospitalized for rhinovirus infection had significantly higher rates of SRF (25.0% vs 13.3%, *P*<0.001), secondary bacterial pneumonia (57.7% vs 39.5%, *P*<0.001) and AKI (33.3% vs 12.6%, *P*<0.001) compared to seasonal influenza (Fig. 1). However, mortality appeared to be lower among adults hospitalized for rhinovirus when compared to influenza (4.3% vs 5.5%, *P*=0.02). The causes of death of the patients in the two groups are summarized in Table S2.

**Fig. 1.**
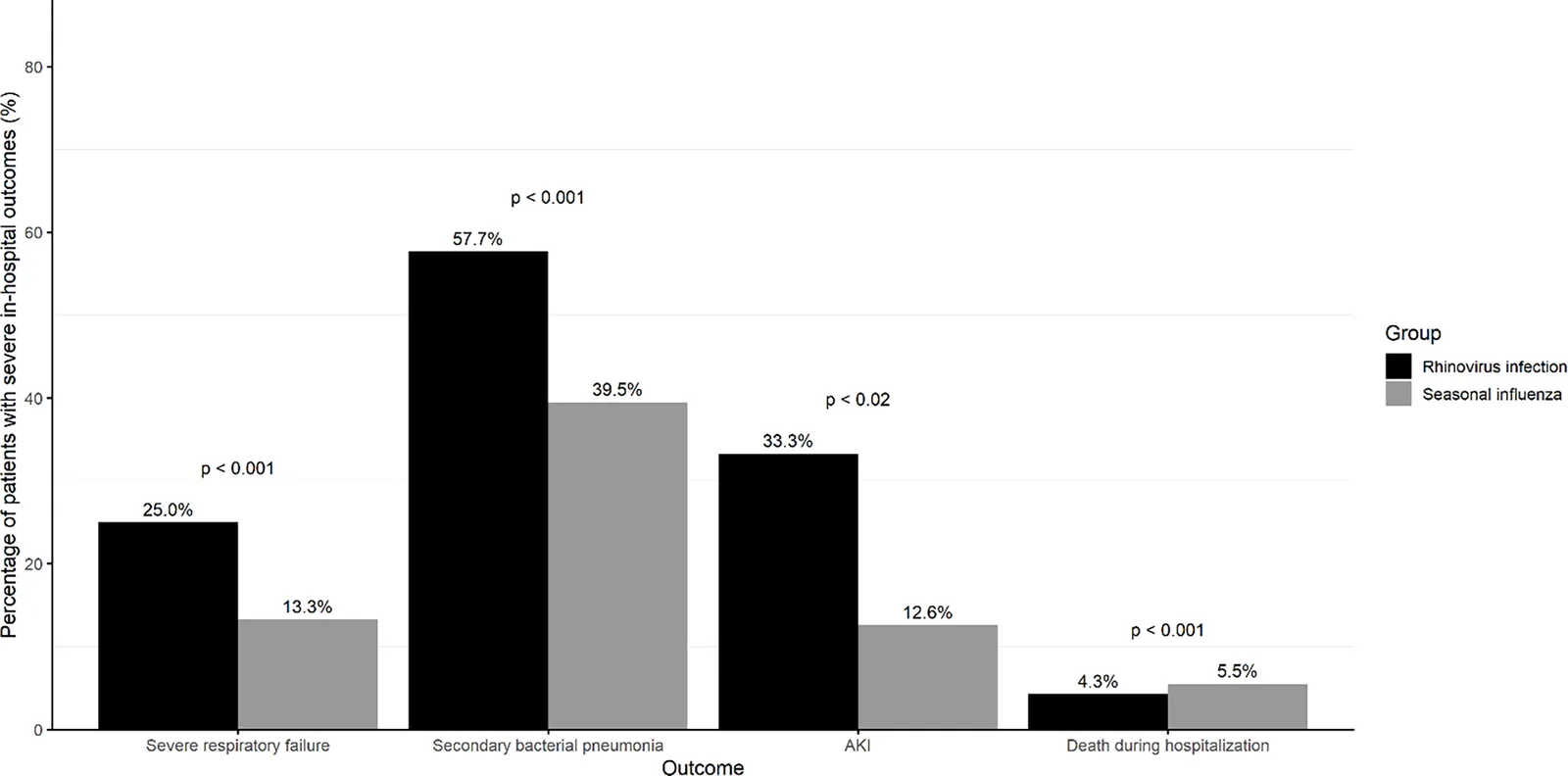
Severe in-hospital adverse outcomes among patients admitted for seasonal influenza or rhinovirus infections among the whole cohort represented in Table 1.

Multivariable analyses demonstrated that rhinovirus infection remained to show higher risk of SRF [adjusted odds ratio (aOR) 1.66; 95% CI 1.47–1.87, *P*<0.001], secondary bacterial pneumonia (aOR 2.01; 95% CI 1.81–2.22, *P*<0.001) and AKI (aOR 1.51; 95% CI 1.35–1.68, *P*<0.001) than seasonal influenza.

The results were largely consistent in the propensity score-matched cohort with the results summarized in Table S3.

### CCI and severe in-hospital outcomes among rhinovirus patients

We assessed the association between CCI as categorical [none (0 points), mild (1–2 points), moderate (3–4 points) and severe (≥5 points)] and a continuous variable. The optimal CCI cut-off for predicting adverse outcomes was determined by ROC analysis (Fig. S1).

Higher grade of CCI was associated with increased risks of all the outcomes. Mortality during index admission was significantly increased among patients with severe comorbidity (CCI≥5), while mild (CCI=1–2) and moderate (CCI=3–4) comorbidity were associated with elevated risks though not reaching statistical significance. For SRF, both moderate and severe degrees of CCI showed escalated risk. Mild, moderate and severe levels of CCI were all associated with increased risk of secondary bacterial pneumonia. Moderate and severe degrees of CCI were also associated with escalated risk of AKI (Figs 2–5 and Table S3).

**Fig. 2.**
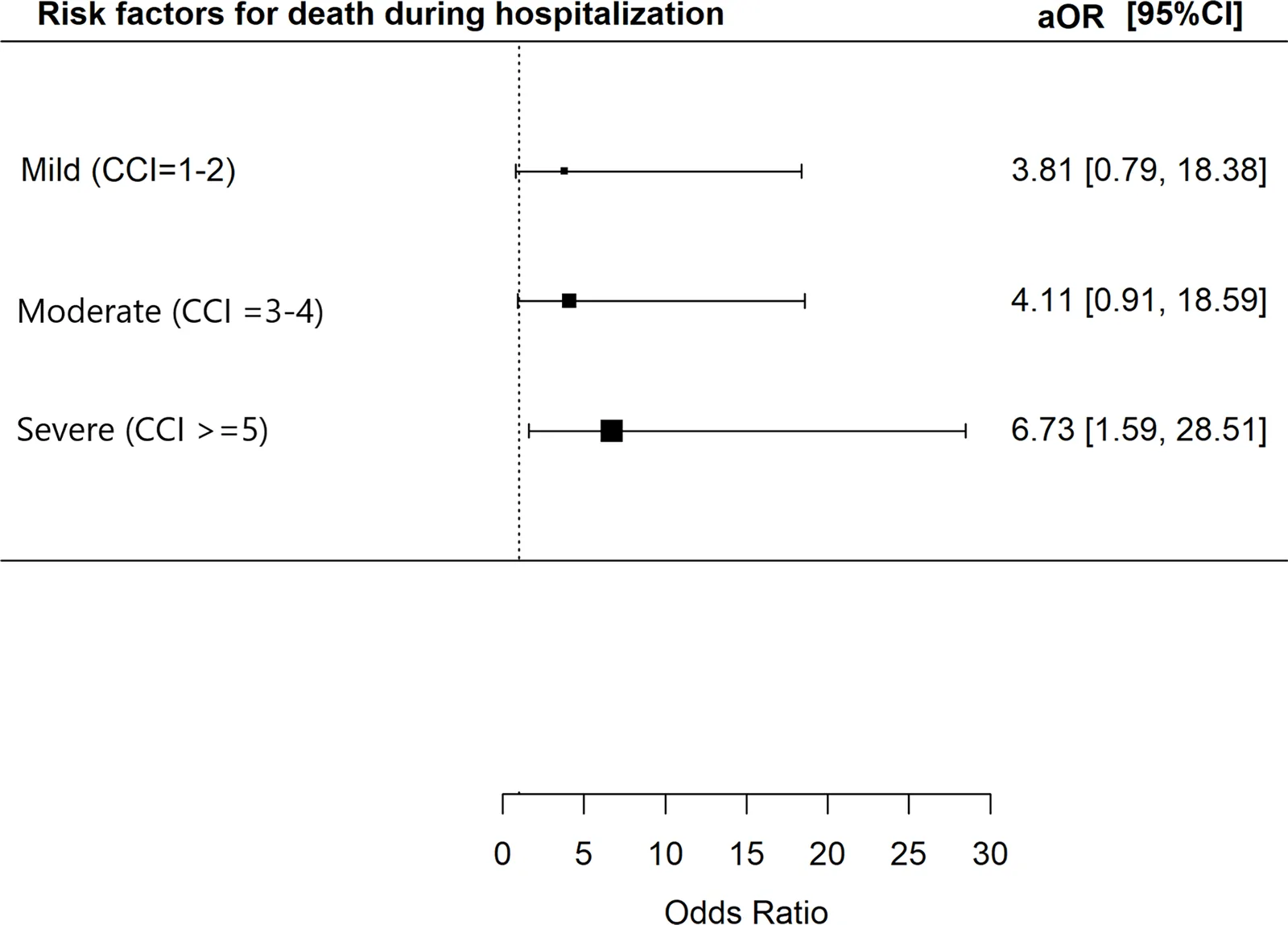
The impact of multiple comorbidities (CCI) on mortality in patients hospitalized for rhinovirus infection.

**Fig. 3.**
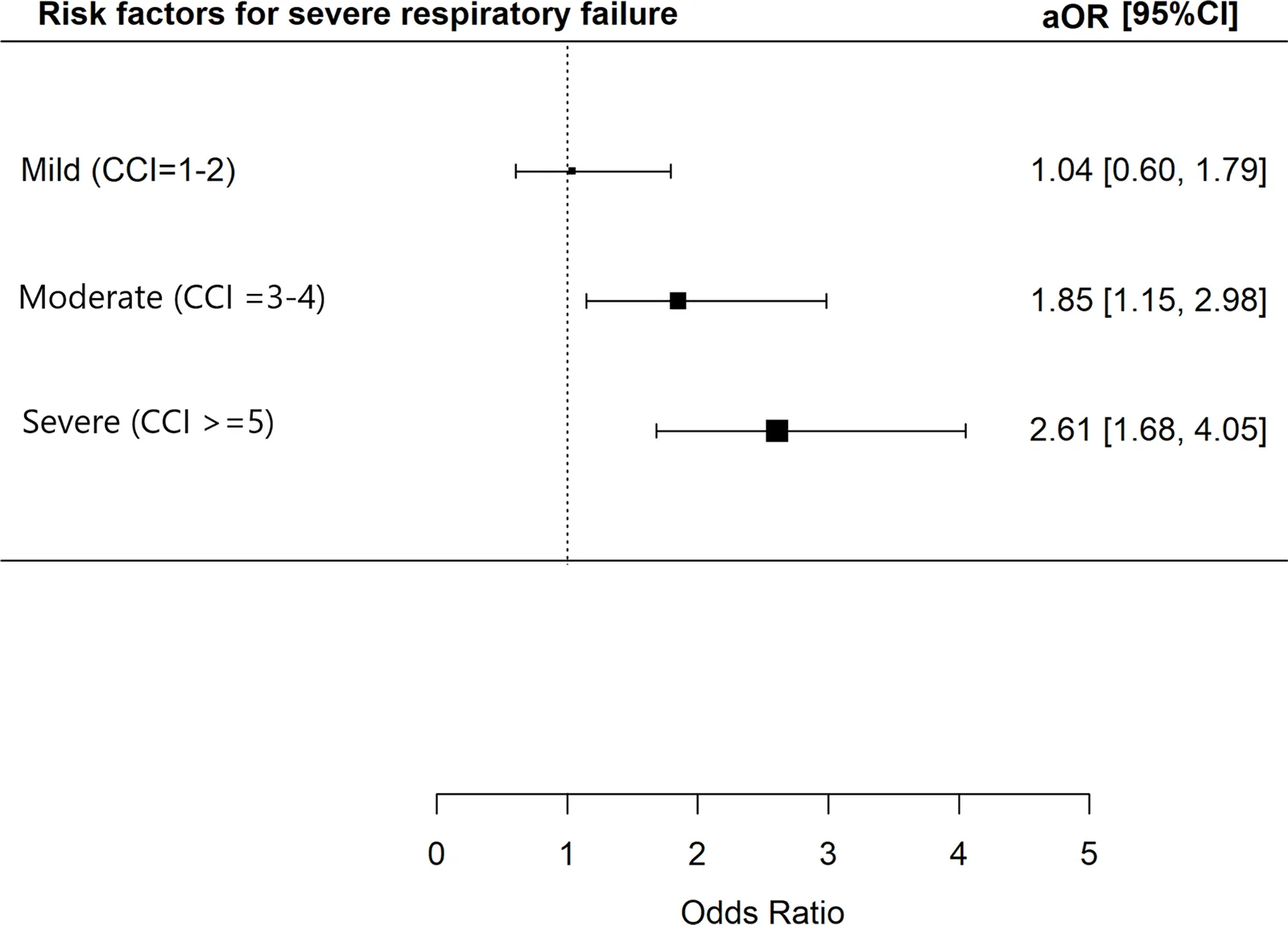
The impact of multiple comorbidities (CCI) on SRF in patients hospitalized for rhinovirus infection.

**Fig. 4.**
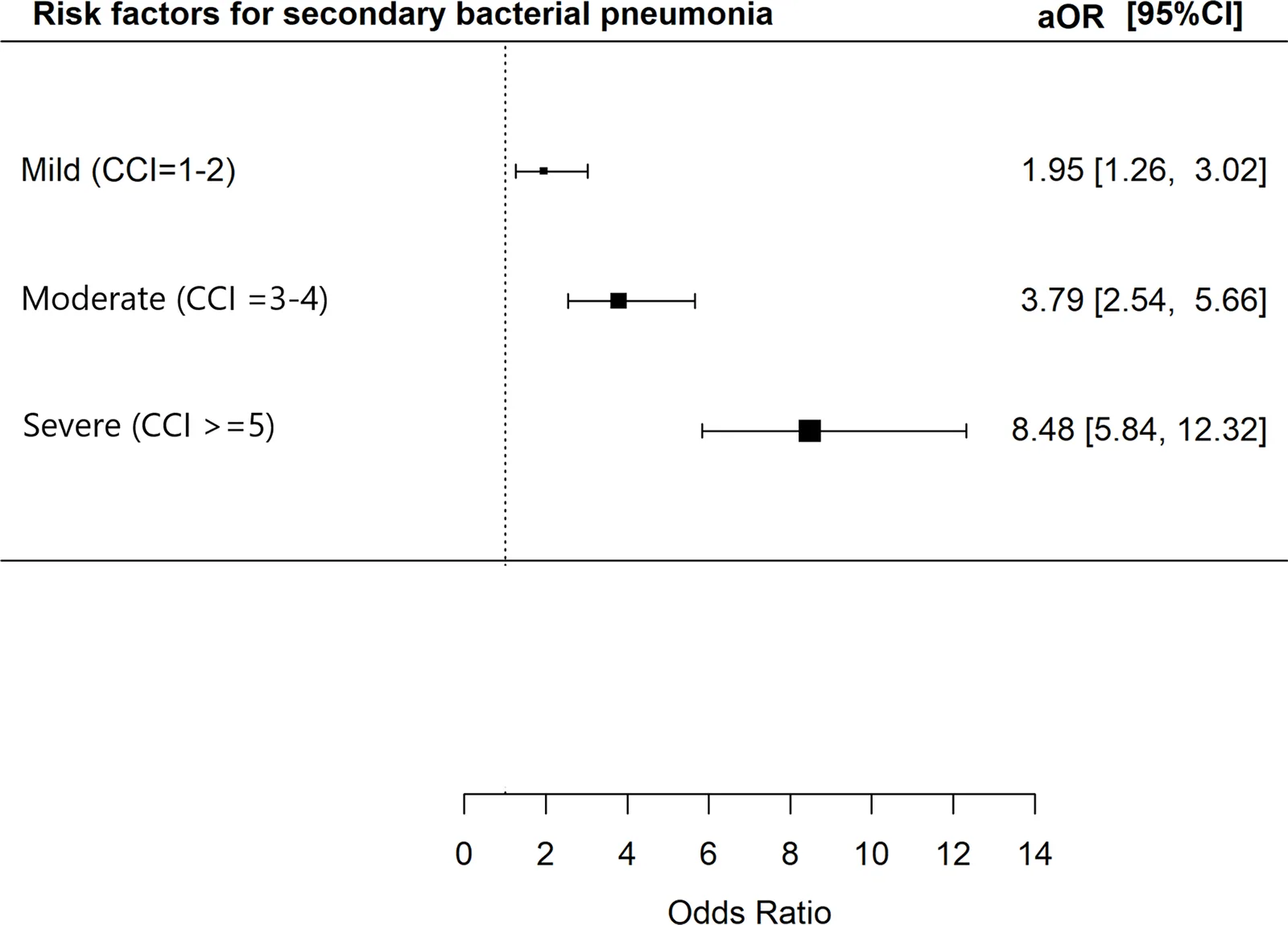
The impact of multiple comorbidities (CCI) on secondary bacterial pneumonia in patients hospitalized for rhinovirus infection.

**Fig. 5.**
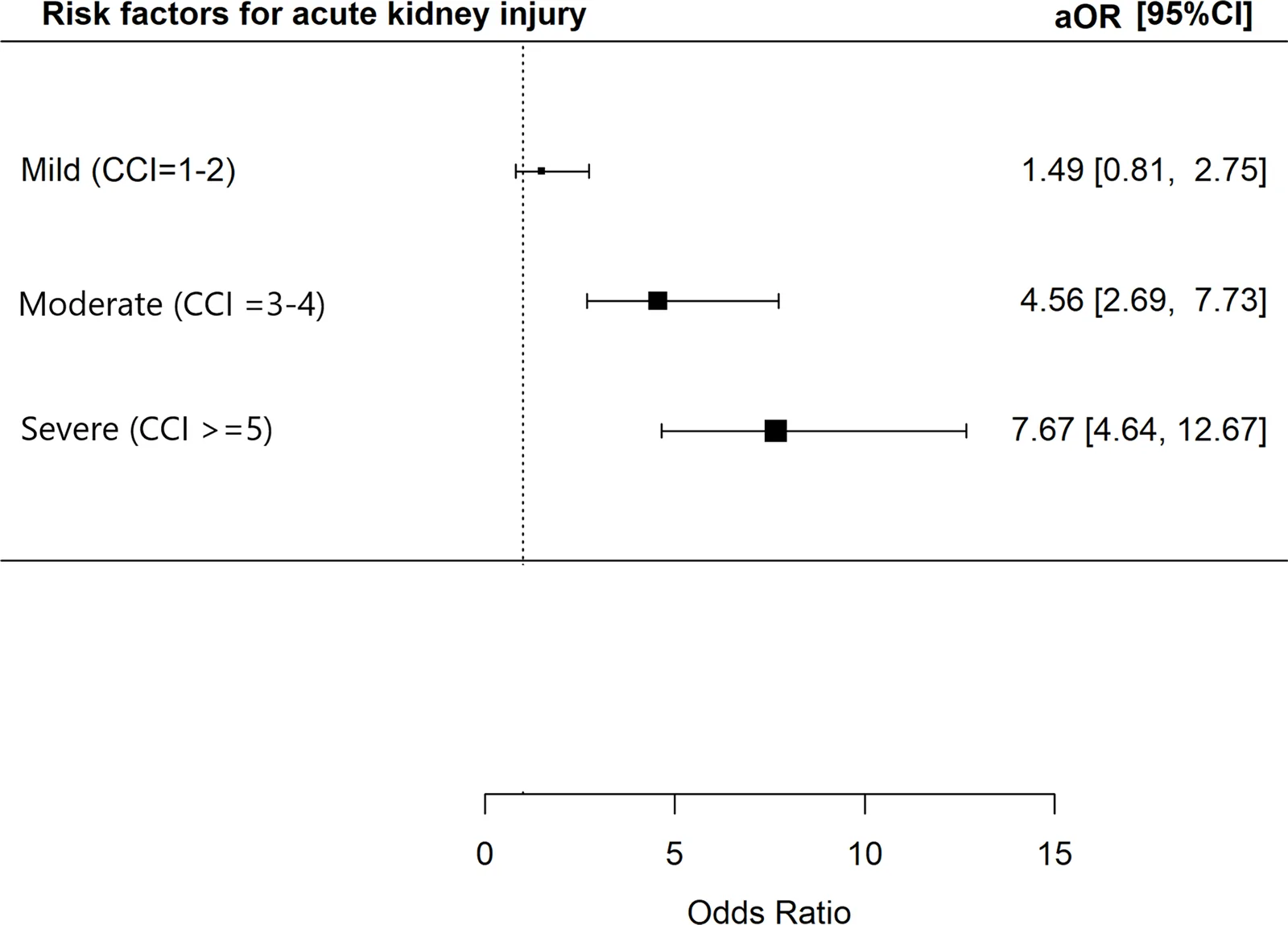
The impact of multiple comorbidities (CCI) on AKI in patients hospitalized for rhinovirus infection.

To define a cut-off for CCI and the development of serious in-hospital outcomes, ROC analysis was performed. At ROC analysis, a CCI cut-off of 3 showed the best performance for predicting a composite outcome of mortality, SRF, secondary bacterial pneumonia and AKI (area under curve 0.72; sensitivity 81.5%, specificity 53.2%, Youden’s index 0.35, 95% CI 0.70–0.75, *P*<0.001). Patients with CCI ≥3 showed significantly increased risks of mortality in index admission, SRF, secondary bacterial pneumonia and AKI, with aOR of 2.41 (95% CI=1.21–4.80, *P*=0.012), 2.35 (95% CI=1.74–3.18, *P*<0.001), 4.54 (95% CI=3.58–5.77, *P*<0.001) and 5.30 (95% CI=3.85–7.30, *P*<0.001), respectively.

When analysed as a continuous variable, CCI remained an important predictor for mortality during index admission, SRF, secondary bacterial pneumonia and AKI (Table S1).

### Subgroup analyses

Consistent with the main analyses, subgroup analyses showed that patients aged <65 years and ≥65 years who were hospitalized for rhinovirus infection, with more comorbidities, as reflected by CCI, were associated with higher risks of severe in-hospital outcomes but not for mortality (Tables S5–6).

## Discussion

Our study showed that adult patients hospitalized for rhinovirus infection were associated with significantly higher risk of adverse in-hospital outcomes than those with seasonal influenza. Multi-morbidities, as reflected by a higher CCI, were also found to be associated with severe in-hospital outcomes among patients hospitalized for rhinovirus infection. These findings can help clinicians to stratify the risk of adult patients hospitalized for rhinovirus infections, using CCI as the readily available and accessible tool.

While rhinovirus can cause asymptomatic and mild infections such as the common cold, severe infections can also occur among hospitalized patients. Unfortunately, there has been a lack of vaccines and anti-viral treatment available for now. With advances in technology and ongoing research, there are possibilities for the development of preventive and therapeutic strategies for rhinovirus infection in the future. For example, there was a recent development of an organoid-based system to reproducibly propagate the poorly cultivable human rhinovirus C (HRV-C), followed by a comprehensive characterization of HRV-C infection and innate immunity in physiologically active respiratory organoids, which can be extended for developing antiviral strategies [17]. Furthermore, advances in RNA therapeutics for respiratory viruses may enable the development of rhinovirus-targeted vaccines and anti-virals [18]. However, since rhinovirus is common, it is important to identify the at-risk group to offer protection. The findings of this study suggested that the CCI can serve as a readily available tool to prognosticate the patients hospitalized for rhinovirus infections. This can also provide hints to clinicians on who should be given priority for rhinovirus vaccinations and anti-virals when they are developed. Patients who are older or have more comorbidities by CCI, especially those with moderate to severe grade (CCI ≥3), are at increased risk of developing respiratory and renal complications. They should be the patients who shall have the priority of preventive and therapeutic options when available.

CCI has been assessed in prognostication for various conditions [19–21], including respiratory tract infections such as COVID-19 [11]. CCI has the advantage of being easy to use and has been well validated in multiple cohorts. It can provide a quick assessment of the risk profile for adult patients hospitalized for rhinovirus infection. It should be considered for use in routine care for adult patients hospitalized for rhinovirus infection for risk stratification. However, as the cut-off of CCI was derived from an exploratory ROC analysis instead of being a pre-specified cut-off, it should be interpreted with the necessary caution.

Another important message from this study is that adult patients hospitalized for rhinovirus infections may face higher risks of serious in-hospital outcomes than those hospitalized for influenza. We demonstrated that the patients hospitalized for rhinovirus infections had higher risks of developing adverse respiratory and renal outcomes, but not mortality. Secondary bacterial pneumonia in patients with rhinovirus infections has been reported. Biomarkers such as myxovirus resistance protein A have also been postulated to cause the secondary bacterial pneumonia [22]. Nasopharyngeal pneumococcal bacterial load was also proposed to be a marker for this [23]. In patients with rhinovirus infections, SRF can result from viral pneumonia [24–26], secondary bacterial pneumonia [27, 28] and also exacerbation of underlying respiratory diseases [29, 30]. The association between rhinovirus infection and AKI includes sepsis [31–33], shock, hypoxemia [34] and even rhabdomyolysis [35]. This should alert clinicians not to underestimate the risks of rhinovirus infections, as previously reported [8]. While the majority of patients may present with a common cold, once hospitalization is required, they are already at risk of developing complications, especially among patients with higher CCI. They shall be offered closer monitoring for the possible development of complications such as secondary bacterial pneumonia, which can be treated with appropriate antimicrobials. Importantly, although rhinovirus was not associated with increased mortality, it can lead to various adverse respiratory and renal complications such as secondary bacterial pneumonia, SRF and AKI. These complications can pose significant healthcare costs and burden, including the use of expensive big-gun antibiotics, ventilatory support, intensive care unit care, renal replacement therapies and prolonged hospitalizations. Of note, despite higher risks of serious in-hospital outcomes among patients hospitalized for rhinovirus infections, these patients did not have higher mortality than those hospitalized for influenza. From our analysis of the causes of death, secondary infections, malignancies and gastrointestinal causes of death were more common among patients with influenza infection. However, there was no significant difference in other causes of death, such as respiratory and cardiovascular causes. As such, the difference in mortality between the two groups needs to be interpreted with caution, as the predominant causes of death were not significantly different between the two groups and the observed difference might have been driven by other causes.

Our study has several limitations. First, we have not analysed the granular details of secondary bacterial infections. Second, data on disease severity scores (e.g. Sequential Organ Failure Assessment Score or Acute Physiology and Chronic Health Evaluation (APACHE)) were not available. The lack of these scores limits the assessment of disease severity at presentation and during the treatment course. As such, we only analysed those available outcomes such as SRF, secondary bacterial pneumonia, AKI and mortality. If these scores had been available, they should be compared between the two groups to represent the severity at presentation and serially compared during the disease course. Nevertheless, our multivariable regression has adjusted for important covariates, and the results appeared to be consistent with our main analysis. Another limitation was the discrepancy between the number of patients in the influenza group and rhinovirus group, which was related to the prevalence of these two viral infections, especially among the hospitalized patients. There were also differences in the clinical characteristics of these patients. While we performed multivariable analyses to adjust for the covariates, residual confounding may still be present. We have also performed sensitivity analyses to ensure our data were robust across different age groups. There was a small amount of missing data in ethnicity (<0.1%), and those subjects with unknown ethnicity were grouped as ‘others’. Furthermore, our data were derived from a territory-wide electronic health record system that captures comprehensive clinical information of all adults hospitalized for rhinovirus or seasonal influenza infection during the study period and is therefore a good representation of real-world data of this clinical entity. As a retrospective study, despite adjusting for important covariates in multivariable analysis, potential confounders may still be present.

## Conclusions

Adults hospitalized for rhinovirus infection were associated with a significantly increased risk of adverse respiratory and kidney outcomes compared with those with seasonal influenza. More medical comorbidities, as reflected by the CCI, were shown to be associated with increased risks of severe in-hospital outcomes.

## Supplementary material

10.1099/jmm.0.002194 Supplementary Material 1.

## Abbreviations

AKI: acute kidney injury
aOR: adjusted odds ratio
APACHE: acute physiology and chronic health evaluation
CCI: Charlson comorbidity index
CDARS: Clinical Data Analysis and Reporting System
COVID-19: coronavirus disease 2019
eGFR: estimated glomerular filtration rate
HA: Hospital Authority
HRV-C: human rhinovirus C
ICD: International Classification of Diseases
IRB: Institutional Review Board
ROC: receiver operating characteristic
SRF: severe respiratory failure
