# Supplementary material for: A territory-wide study on adults hospitalized for rhinovirus infection – mortality, severe adverse respiratory and renal outcomes and relationships with multiple comorbidities
Source: J Med Microbiol. 2026 Oct 7;75(10):002194. doi: 10.1099/jmm.0.002194 (PMC13645376; doi:10.1099/jmm.0.002194)

Supplementary Table 1 Multicollinearity testing of the covariates adjusted in multivariable analyses

Covariates	Variance Inflation Factor (VIF)
Sex	1.03
Presence of chronic airway diseases	1.08
Influenza vaccine	1.33
pneumococcal polysaccharide vaccine	1.14
Pneumococcal conjugated vaccine	1.30

Supplementary Table 2 Primary cause of death among patients hospitalized for seasonal influenza or rhinovirus infections

	**Seasonal influenza (n = 2266)**	**Rhinovirus infection (n = 96)**	**p-value**
**Respiratory cause**	1486	73	0.37
**Cardiovascular cause**	242	8	0.16
**Secondary infection**	42	8	0.01
**Underlying viral infection**	4	1	0.67
**Malignancies**	232	3	0.01
**Renal causes**	109	3	0.23
**Gastrointestinal causes**	60	0	0.07
**Central nervous system cause**	18	0	0.32
**Other causes**	33	0	0.14

Supplementary Table 3 Severe in-hospital outcomes among influenza and rhinovirus patients in propensity score matched cohort

Outcomes	Patient groups	Number of subjects (%)	Univariate analysis			Multivariate† analysis		
			OR	95 % CI	p-value	aOR	95 % CI	p-value
Death during hospitalization*	Seasonal influenza	154(4.50)	-Reference-			-Reference-		
	Rhinovirus	83(4.43)	0.983	0.748-1.292	0.902	0.980	0.744-1.291	0.886
Severe respiratory failure*	Seasonal influenza	696(20.37)	-Reference-			-Reference-		
	Rhinovirus	454(24.25)	1.252	1.094-1.432	0.001	1.235	1.074-1.419	0.003
Secondary bacterial pneumonia *	Seasonal influenza	1777(52.00)	-Reference-			-Reference-		
	Rhinovirus	1021(54.54)	1.107	0.989-1.24	0.077	1.112	0.985-1.254	0.085
AKI*	Seasonal influenza	704(20.60)	-Reference-			-Reference-		
	Rhinovirus	591(31.57)	1.778	1.564-2.021	<0.001	1.852	1.621-2.115	<0.001

*: Factors that are statistically significant after adjustment for confounders

†: Adjusted for age, sex; CCI, presence of DM, chronic airway diseases, cardio-/cerebro-vascular diseases, and previous vaccination against influenza and pneumococcal polysaccharide and conjugated vaccine.

RSV = Respiratory syncytial virus; CI = Confidence interval; HAP = Hospital acquired pneumonia; AKI = Acute kidney injury

Supplementary Table 4 Association between CCI and severe in-hospital outcomes among rhinovirus patients in whole cohort

	**Univariate analysis**			**Multivariate analysis**		
**Death during hospitalization**						
	**OR**	**95% CI**	**p-value**	**aOR**	**95% CI**	**p-value**
**CCI (Continuous variable)**	1.07	0.99 – 1.15	0.055	1.08	1.01 – 1.16	0.03
**CCI grade**						
**No**	Reference group					
**Mild**	3.41	0.72 – 16.24	0.12	3.81	0.79 - 18.39	0.010
**Moderate**	3.67	0.83 – 16.20	0.09	4.11	0.91 - 18.61	0.07
**Severe**	5.63	1.37 – 23.13	0.02	6.73	1.59 - 28.53	0.01
**CCI ≥ 3**	2.21	1.14 - 4.29	0.02	2.41	1.21 – 4.80	0.01
**SRF**						
	**OR**	**95% CI**	**p-value**	**aOR**	**95% CI**	**p-value**
**CCI (Continuous variable)**	1.11	1.07 – 1.15	<0.001	1.11	1.07 – 1.15	<0.001
**CCI grade**						
**No**	Reference group					
**Mild**	1.12	0.66 – 1.90	0.68	1.04	0.601 - 1.78	0.90
**Moderate**	1.82	1.15 – 2.88	0.011	1.85	1.15 - 2.99	0.01
**Severe**	2.61	1.73 – 3.96	<0.001	2.61	1.683 - 4.05	<0.001
**CCI ≥ 3**	2.25	1.70 – 2.99	<0.001	2.53	1.74 – 3.18	<0.001
**Secondary bacterial pneumonia**						
	**OR**	**95% CI**	**p-value**	**aOR**	**95% CI**	**p-value**
**CCI (Continuous variable)**	1.30	1.25 – 1.34	<0.001	1.30	1.25 – 1.34	<0.001
**CCI grade**						
**No**	Reference group					
**Mild**	2.05	1.33 – 3.15	0.001	1.95	1.26 - 3.02	0.003
**Moderate**	4.04	2.74 – 5.96	<0.001	3.79	2.544 - 5.66	<0.001
**Severe**	8.80	6.15 – 12.59	<0.001	8.48	5.84 – 12.32	<0.001

**CCI ≥ 3**	4.72	3.76 – 5.92	<0.001	4.54	3.58 – 5.77	<0.001
**AKI**						
	**OR**	**95% CI**	**p-value**	**aOR**	**95% CI**	**p-value**
**CCI (Continuous variable)**	1.22	1.18 – 1.26	<0.001	1.22	1.18 – 1.26	<0.001
**CCI grade**						
**No**	Reference group					
**Mild**	1.41	0.77 – 2.58	0.26	1.49	0.81 - 2.75	0.198
**Moderate**	4.20	2.51 – 7.03	<0.001	4.56	2.69 - 7.73	<0.001
**Severe**	6.97	4.29 – 11.31	<0.001	7.67	4.64 - 12.66	<0.001
**CCI ≥ 3**	5.07	3.72 – 6.91	<0.001	5.30	3.85 – 7.30	<0.001

Supplementary Table 5 Association between CCI and severe in-hospital outcomes among rhinovirus patients age < 65 years (n = 669)

	**Univariate analysis**			**Multivariate analysis**		
**Death during hospitalization**						
	**OR**	**95% CI**	**p-value**	**aOR**	**95% CI**	**p-value**
**CCI (Continuous variable)**	1.09	0.87 – 1.37	0.46	1.07	0.84 – 1.35	0.61
**CCI grade**						
**No**	Reference group					
**Mild**	2.82	0.56 – 14.13	0.21	2.76	0.55 - 14.01	0.22
**Moderate**	0	0 - 0	1.00	0	0 - 0	1.00
**Severe**	3.96	0.71 – 21.99	0.12	3.02	0.49 – 18.58	0.23
**CCI ≥ 3**	0.87	0.26 – 2.91	0.82	0.74	0.19 – 2.88	0.67
**SRF**						
	**OR**	**95% CI**	**p-value**	**aOR**	**95% CI**	**p-value**
**CCI (Continuous variable)**	1.16	1.07 – 1.25	<0.001	1.11	1.10– 1.22	<0.001
**CCI grade**						
**No**	Reference group					
**Mild**	1.18	0.69 – 2.01	0.56	1.02	0.58 – 1.80	0.95
**Moderate**	1.59	0.90 – 2.83	0.11	1.46	0.79 – 2.70	0.23
**Severe**	2.95	1.73 – 3.96	<0.001	2.29	1.22 - 4.29	0.01
**CCI ≥ 3**	1.95	1.32 – 2.89	<0.001	1.80	1.15 – 2.74	0.01
**Secondary bacterial pneumonia**						
	**OR**	**95% CI**	**p-value**	**aOR**	**95% CI**	**p-value**
**CCI (Continuous variable)**	1.26	1.17 – 1.35	<0.001	1.21	1.12 – 1.31	<0.001
**CCI grade**						
**No**	Reference group					
**Mild**	1.93	1.24 – 3.00	0.003	1.66	1.05 - 2.62	0.03
**Moderate**	3.32	2.06 – 5.34	<0.001	2.64	1.60 - 4.36	<0.001
**Severe**	4.13	2.48 – 6.89	<0.001	3.08	1.78 – 5.31	<0.001
**CCI ≥ 3**	2.53	1.82 – 3.51	<0.001	2.11	1.48 – 3.00	<0.001
**AKI**						
	**OR**	**95% CI**	**p-value**	**aOR**	**95% CI**	**p-value**

**CCI (Continuous variable)**	1.22	1.12 – 1.32	<0.001	1.24	1.13 – 1.35	<0.001
**CCI grade**						
**No**	Reference group					
**Mild**	1.52	0.83 – 2.80	0.18	1.64	0.88 - 3.03	0.12
**Moderate**	3.17	1.72 – 5.83	<0.001	3.28	11.74 – 6.19	<0.001
**Severe**	4.19	2.23 – 7.85	<0.001	4.68	22.40 – 9.13	<0.001
**CCI ≥ 3**	2.84	1.88 – 4.28	<0.001	2.87	1.86 – 4.43	<0.001

Supplementary Table 6 Association between CCI and severe in-hospital outcomes among rhinovirus patients age ≥ 65 years (n = 1553)

	**Univariate analysis**			**Multivariate analysis**		
**Death during hospitalization**						
	**OR**	**95% CI**	**p-value**	**aOR**	**95% CI**	**p-value**
**CCI (Continuous variable)**	0.94	0.85 – 1.04	0.27	0.95	0.86 – 1.06	0.36
**CCI grade**						
**Mild**	Reference group					
**Moderate**	0.6	0.13 – 2,80	0.52	0.52	1.11 – 2.50	1.00
**Severe**	0.63	0.15 – 2.73	0.54	0.59	0.13 – 2.64	0.49
**CCI ≥ 3**	0.62	0.14– 2.67	0.82	0.74	0.19 – 2.88	0.67
**SRF**						
	**OR**	**95% CI**	**p-value**	**aOR**	**95% CI**	**p-value**
**CCI (Continuous variable)**	1.08	1.02 – 1.13	0.004	1.11	1.10 – 1.22	<0.001
**CCI grade**						
**Mild**	Reference group					
**Moderate**	3.39	0.78 – 114.76	0.11	1.46	0.79 – 2.70	0.23
**Severe**	2.95	1.73 – 3.96	0.04	2.29	1.22 - 4.29	0.01
**CCI ≥ 3**	4.28	1.00 – 18.30	0.05	4.82	1.11 – 20.91	0.004
**Secondary bacterial pneumonia**						
	**OR**	**95% CI**	**p-value**	**aOR**	**95% CI**	**p-value**
**CCI (Continuous variable)**	1.21	1.15 – 1.27	<0.001	1.21	1.12 – 1.31	<0.001
**CCI grade**						
**Mild**	Reference group					
**Moderate**	1.37	0.59 – 3.15	0.46	1.35	0.56 – 3.22	0.50
**Severe**	2.89	1.28 – 6.52	0.01	3.01	1.29 – 7.03	0.01
**CCI ≥ 3**	2.48	1.10 – 5.57	0.03	2.55	1.10 – 5.93	0.03
**AKI**						
	**OR**	**95% CI**	**p-value**	**aOR**	**95% CI**	**p-value**
**CCI (Continuous variable)**	1.13	1.08 – 1.19	<0.001	1.24	1.13 – 1.35	<0.001
**CCI grade**						
**Mild**	Reference group					

**Moderate**	11.21	1.49 – 84.22	0.02	10.83	1.44 – 81.53	0.021
**Severe**	17.14	2.31 – 127.29	0.01	16.60	2.23 – 123.50	0.01
**CCI ≥ 3**	15.86	2.14 – 1117.73	0.01	15.35	2.06 – 114.41	0.01

Supplementary Figure 1 Receiver operating characteristic curve for CCI and composite severe in-hospital outcomes severe

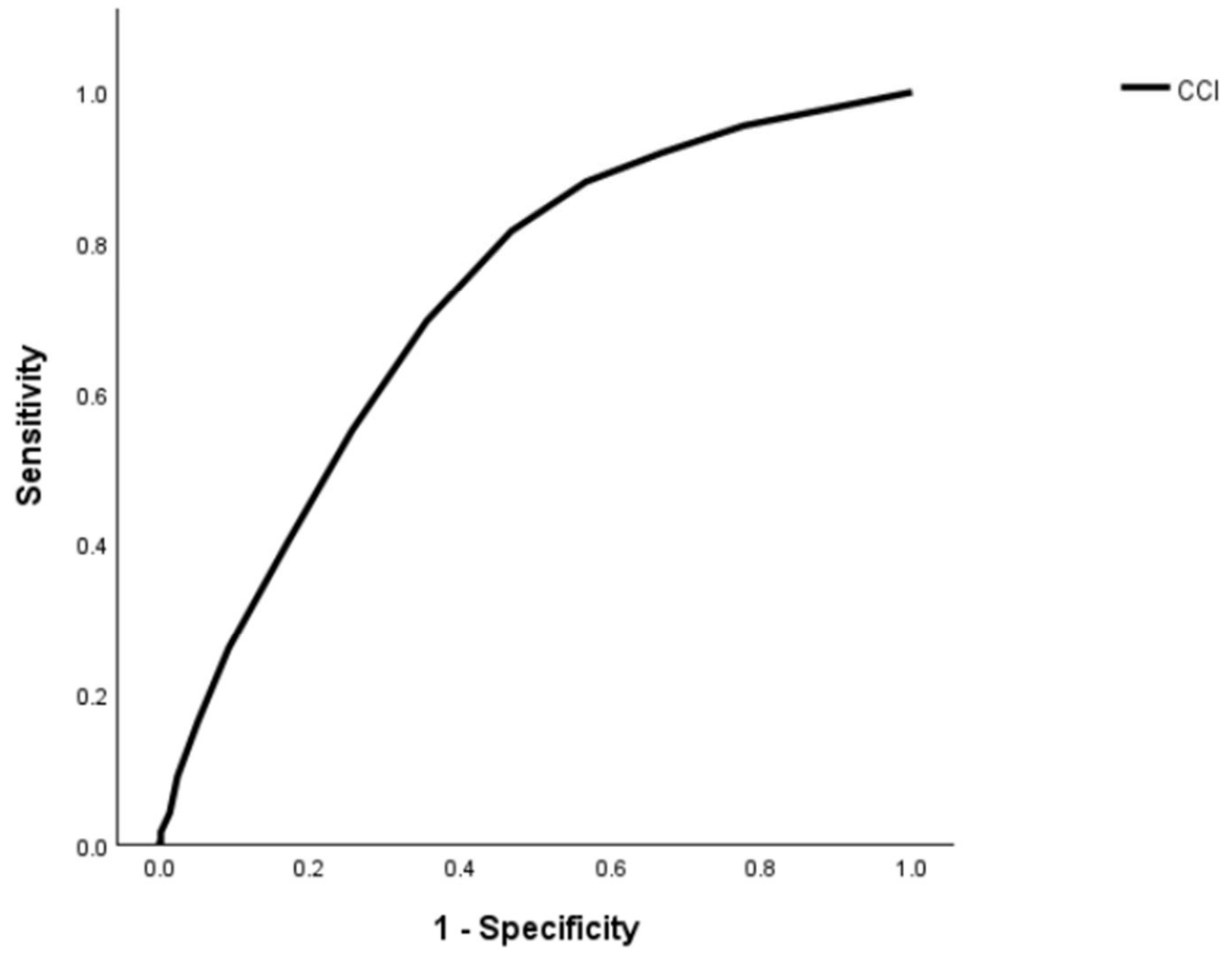

Supplement: Supplementary file 1 [file jmm-75-02194-s001.pdf]
